# Plasminogen Activator Inhibitor-1 and Oncogenesis in the Liver Disease

**DOI:** 10.33696/signaling.2.054

**Published:** 2021

**Authors:** Da-eun Nam, Hae Chang Seong, Young S. Hahn

**Affiliations:** 1 Beirne B. Carter Center for Immunology Research, University of Virginia, Charlottesville, USA; 2 Department of Microbiology, Immunology, and Cancer Biology, University of Virginia, Charlottesville, USA

**Keywords:** PAI-1, CSCs, HCV, Hepatocytes, HCC

## Abstract

Hepatocellular carcinoma (HCC) is a significant cause of cancer mortality worldwide. Chronic hepatic inflammation and fibrosis play a critical role in the development of HCC. Liver fibrosis develops as a result of response to injury such that a persistent and excessive wound healing response induces extracellular matrix (ECM) deposition leading to HCC. PAI-1 is a fibrinolysis inhibitor involved in regulating protein degradation and homeostasis while assisting wound healing. PAI-1 presents increased levels in various diseases such as fibrosis, cancer, obesity and metabolic syndrome. Moreover, PAI-1 has been extensively studied for developing potential therapies against fibrosis. In the present review, we summarize how PAI-1 affects oncogenesis during liver disease progression based on the recently published literatures. Although there are controversies regarding the role of PAI-1 and approaches to treatment, this review suggests that proper manipulation of PAI-1 activity could provide a novel therapeutic option on the development of chronic liver disease via modulation of cancer stem-like cells (CSCs) differentiation.

## Plasminogen Activator Inhibitor-1 (PAI-1) Plays a Major Role in Regulating Protein Synthesis and Wound Healing Process

**Figure 1:**
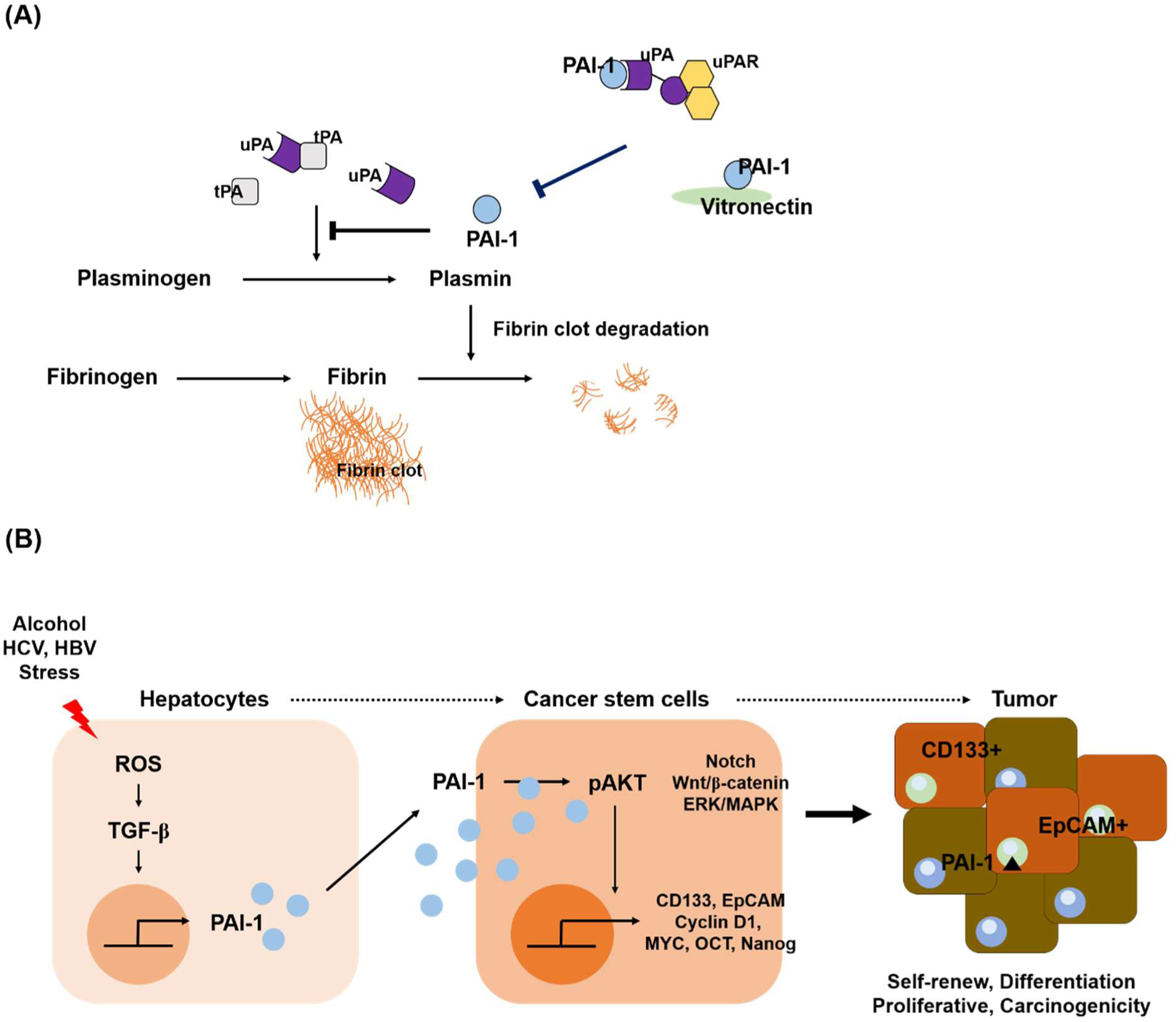
Schematic showing regulation of PAI-1 activation and its role in hepatocytes. **(A)** Plasminogen pathway and PAI-1 regulation in normal condition. **(B)** Hepatic injury caused by alcohol, HCV, stress, and a high-fat diet, stimulates TGF-β synthesis leading to PAI-1 expression in hepatocytes. The increase in PAI-1 level induces AKT activation and promotes differentiation into cancer stem-like cells (CSC) with characteristics such as self-renewal, cell proliferation and cell survival. Differentiation into CSC is accompanied by increased expression of numerous oncogenes such as Cyclin D1, MYC, OCT, and Nanog, and ultimately plays a key role in the progression of HCC.

The plasminogen pathway regulates the homeostasis of ECM structures through fibrinolysis. Plasminogen is converted to plasmin by plasminogen activators (PAs): tissue-type PA (tPA) and urokinase-type PA (uPA) in various tissues, leading to proteolysis. Plasminogen activator inhibitor-1 (PAI-1) is a major regulator of the plasminogen pathway and is involved in regulating the tPA/uPA activity (Figure 1A). PAI-1 is a member of the serine protease inhibitor gene family, and is mainly produced by the endothelium and is expressed on various cell types such as adipocytes, macrophages, cardiomyocytes, and fibroblasts. PAI-1 gene expression is affected by numerous transcription factors and cell types, and is closely regulated by cytokines and growth factors including transforming growth factor-β (TGF-β), interleukin-1β (IL-1β), epidermal growth factor (EGF), and insulin. Specifically, injured cells undergo oxidative stress in response to various damage and induce TGF-β to activate fibroblast. Thereby TGF-β promotes the expression of PAI-1 and collagen genes contributing to develop fibrogenesis. Under normal condition, PAI-1 plays a role in the wound repair process by ECM protein deposition via inhibiting the activity of uPA/tPA/plasmin-dependent MMPs. This activity can be regulated by the formation of the trimeric PAI-1/uPA/uPAR complex or by binding to vitronectin (Figure 1A). However, sustained PAI-1 activity induces excessive accumulation of proteins which leads to an epithelial-mesenchymal transition (EMT) state as well as an increase in cell invasiveness that contributes to fibrosis (Figure 1B). It suggests that excessive PAI-1 activity promotes fibrosis development and thereby may facilitate progression of HCC. However, underlying mechanisms for the pathogenic role of PAI-1 in fibrosis are still unclear. Our recent report demonstrates a correlation between the increase of PAI-1 and CSC differentiation under pathological conditions caused by HCV. These results suggest a potential role for PAI-1 in liver disease progression [1].

## Pathogenic Role of PAI-1 in Chronic Liver Diseases

Abnormal wound healing response under the pathological condition leads to excessive accumulation of ECM proteins in the wound area, and is characterized by aggressive cell growth, hardening and scarring. Fibrosis is defined as a persistent and abnormal wound healing response that occurs while damaged cells are replaced. Therefore, a mechanism of liver tumorigenesis has been proposed based on its ability to induce tissue damage and proliferative repair. PAI-1, involved in collagen synthesis and ECM protein homeostasis, has been suggested as biomarker and a potential therapeutic target in the progression of fibrotic diseases, including cirrhosis and HCC. PAI-1 not only contributes to the fibrinolysis process, but also triggers signal transduction for cell transition, and cell migration in liver tissue [2,3]. The major profibrotic cytokine TGF-β induces PAI-1 expression, and increases collagen and ECM protein synthesis through activation of hepatic stellate cells (HSC). Reports on TGF-β synthesis stimulated by exogenously delivered PAI-1 could support the presence of PAI-1/TGF-β-positive feedback mechanism [4]. Recent study reported that increased PAI-1 level plays a crucial role in angiogenesis and tumor growth induced by TGF-β stimulation in the endothelium [5], and showed that increased PAI-1 along with TGF-β exacerbated fibrosis in mouse models [6]. Genetic deletion of PAI-1 attenuates high-fat diet induced hepatic steatosis [7]. Oral administration of the PAI-1 inhibitor TM5275 attenuates liver fibrosis under the metabolic syndrome in mice [8]. Moreover, there was a significant increase in PAI-expression in chronic HCV patients with HCC/cirrhosis [9]. Direct-acting antiviral (DAA) therapy resulted in a significant decrease in serum PAI-1 from patients with HCV related chronic liver disease compared to chronic HCV patients without treatment [10]. Notably, a recent study identified that an increase in PAI-1 by HCV infection induces AKT activation, which can promote differentiation into cancer stem-like cells (CSC) from hepatocytes [1]. These results provide strong evidence that increased PAI-1 in liver disease may further exacerbate the progression to HCC by engaging in differentiation of the CSC phenotype.

## Liver Cancer Stem-like Cells

CSC are known to be cancer-propagating cells involved in tumor metastasis as they are characterized by self-renewal, cell proliferation and cell survival. CSC have been identified in several tumors such as liver, lung, breast, and pancreatic cancers [11,12]. Several recent studies strongly supported the role of CSC as specialized cells in initiating, maintaining, and giving anticancer drug resistance in liver tumors. Recent studies have identified the role of the CSC as well as their specific markers in the liver disease progression [11], and further on it has been confirmed that increased CSC properties increased EMT from HCV-infected hepatocytes [13–15]. Studies are still ongoing on investigating CSC differentiation and characteristics in liver disease to elucidate clear mechanisms involved in the progression into CSC state. Human Differentiated Protein 133 (CD133), EpCAM, CD44, and CD90 are known as representative markers of a CSC state in various tumor tissues, including HCC [16–18]. CD133+ liver cancer cells showed more powerful capacity in proliferation than CD133− liver cancer cells, and higher levels of pAKT were identified in CD133+ cancer cells than in CD133− cancer cell [19–21]. The PI3K/AKT pathway may be involved in activation through the interaction between Src (Protooncogene tyrosine-protein kinase) of CD133 and P85 [22,23]. Epithelial cell adhesion molecule (EpCAM) is a single transmembrane glycoprotein overexpressed in various tumor types, and is also known as a representative marker of CSC status in liver cancer [24,25]. EpCAM induces cell plasticity within epithelial tissues by weakening cadherin-mediated cell adhesion, which can promote cell proliferation and motility during tumor progression [26]. Moreover, excessive EpCAM expression induces cell proliferation with c-MYC known as a carcinogenic transcription factor. Cell cycle protein Cyclin D1 and pluripotency markers such as Octamer 4 (Oct4) and Nanog, are also reported to be positively correlated with EpCAM [27,28]. Notably, EpCAM-positive cells isolated from HCC patients showed strong CSC features like differentiation and self-renewal properties [16,29]. We observed that HCV-associated elevation of PAI-1 levels induces AKT activation, leading to upregulation of CSC markers including CD133, EpCAM, and oncogenes (eg, MYC, Nanog, OCT, and Cyclin D1) in hepatocytes (Figure 1B). Importantly, inhibition of PAI-1 expression interfered with the progression of the CSC state and AKT activation in HCV-infected hepatocytes [13]. These results suggest that HCV infection in hepatocytes increases cell invasiveness and mobility by stimulating AKT activation through PAI-1 upregulation. It implicates that the manipulation of PAI-1 activity could provide potential therapeutics to prevent the development of HCV-associated chronic liver diseases.

## Therapeutic Strategy

Regulation of protein synthesis is a key system for maintaining cellular homeostasis, therefore abnormal protein synthesis/degradation is inevitably linked to diseases such as thrombosis, fibrosis, and cancer. Numerous studies demonstrate that PAI-1 is significantly elevated in most human cancer. The increased PAI-1 level is positively correlated with poor clinical outcome in patients with breast, ovarian, non-small cell lung cancers, and HCC [30–34]. Particularly, PAI-1 level is significantly elevated in plasma from patients with cirrhosis and hepatocellular carcinoma following HCV infection compared to that of uninfected healthy donors [10,35]. Given this clinical importance of PAI-1 levels related to severity of human diseases, inhibitors have been synthesized and some inhibitors are under clinical trials in various diseases including ovarian cancer, alzheimer’s disease, cardiovascular disease, pulmonary fibrosis renal diseases and liver diseases [36–39].

The most notable drugs are tiplaxtinin (PAI-039) and TM series. Tiplaxtinin has not been successful in human clinical trials because of its unfavorable risk-benefit ratio, potential for bleeding disorders, and the need for stringent dose control. However, both drugs are still widely used in preclinical research and there have been efforts to develop new drugs that share the same mechanism of action. PAI-1 levels are elevated in various diseases and is associated with a poor prognosis [40–44], whereas in some models of plasminogen system deficiency it has reported the opposite effect [45–47)]. For instance, human SERPINE1 mutations caused bleeding disorders due to poor blood coagulation [48]. In contrast, complete inactivation of SERPINE1 mutations reduced the prevalence of metabolic diseases and contributed to increased survival rate [49]. Additionally, while an increase in serum PAI-1 levels has a positive correlation between mortality rates in HCV-infected liver cirrhosis/HCC patients [10,50], other study suggested that HCV viruses may downregulate PAI-1 expression to promote replication [45]. Although the ultimate clinical goal is to treat or prevent disease by modulating PAI-1 activity, these opposing results raise concern that excessive control of PAI-1 may lead to negative consequences on disease progression.

**Figure 2:**
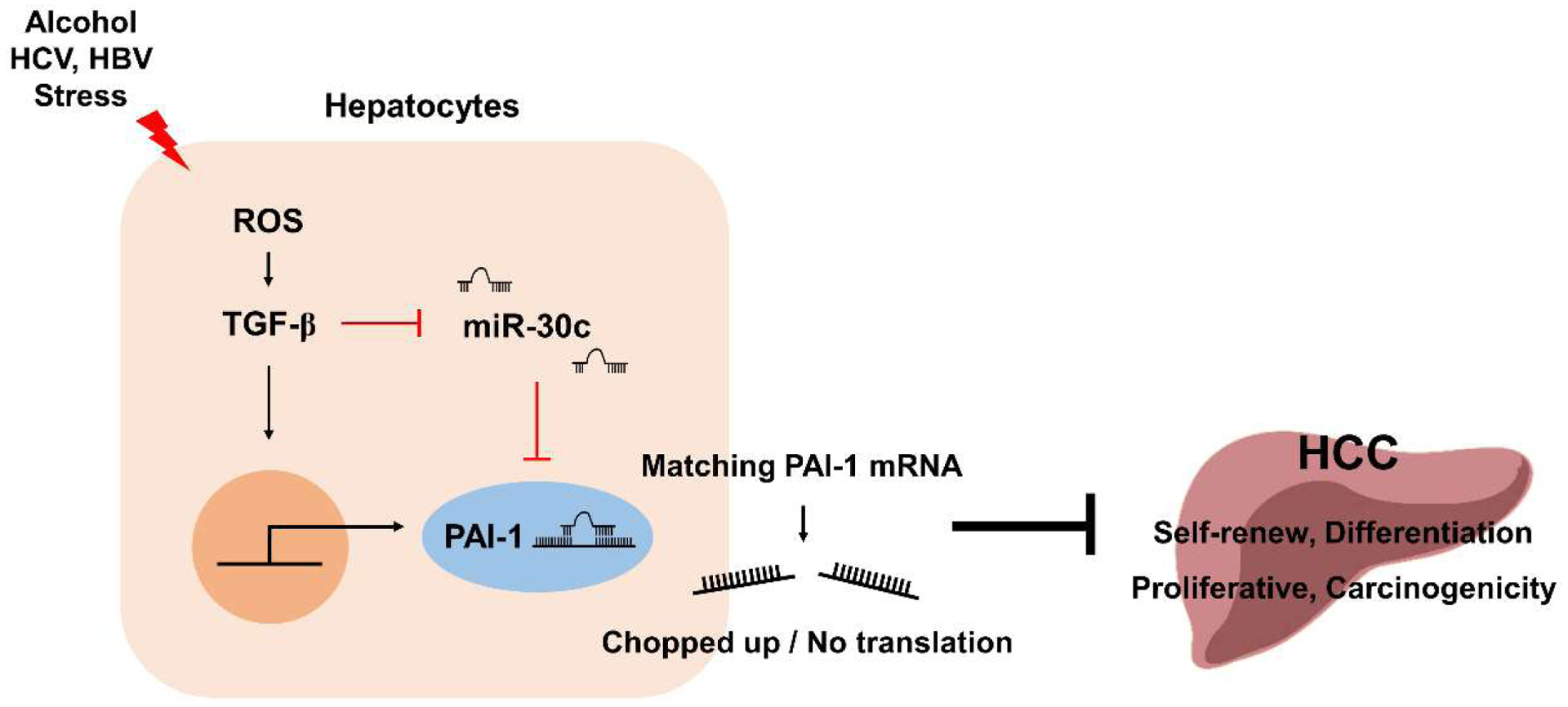
Schematic diagram showing the potential for therapeutic drug development using the pathway to inhibit PAI-1 expression by miR-30c.

Targeting PAI-1 can be subtle since PAI-1 is involved in extensive biological processes. Most of PAI-1 inhibitors act in a reversible manner, either by directly blocking the PAI-1 active domain or the docking site of PAI-1 thereby eliminating or converting tPA/uPA activity to inactive PAI-1 [51,52]. Thus, it would be worthwhile to develop potential drugs targeting PAI-1-associated signaling pathways and/or human-derived PAI-1 regulators like microRNAs. Hepatocyte differentiation into CSC and its related signaling pathway including PI3K/AKT, Wnt/β-catenin, MAPK/ERK, ROS, Notch, and TGF-β, may provide insight on the development of therapeutic agents targeting PAI-1. Furthermore, microRNAs can be considered as another approach to manipulate the expression of PAI-1. Previous studies have reported that microRNA-30c (miR-30c) is involved in the differentiation and activation of hepatocytes and HSC. miR-30c can downregulates PAI-1 expression by binding directly to the 3’-UTR. In particular, it has been reported that miR-30c plays a role in inhibiting collagen synthesis in fibroblasts [53, 54], and TGF-β downregulates miR-30c expression in endothelial cells and HSC [5,55]. Interestingly we observed that inhibition of PAI-1 expression by miR-30c interfered the differentiation into CSC and AKT activation in HCV-infected hepatocytes [13]. This suggests that controls other factors related to PAI-1 regulation may be a more promising strategy, given the limitations on the use of PAI-1 targeted drugs in clinical trials (Figure 2).

## Conclusions

PAI-1 has been studied to understand its pathogenic role in fibrosis and to use it as a therapeutic strategy. However, some conflicting results raise major risks and challenges for developing PAI-1-based new drugs. In particular, the instability and short half-life of PAI-I active form is problematic for exerting therapeutic effects in chronic diseases. Moreover, potential effects of PAI-1 inhibitors on hemostasis make therapeutic approaches difficult. These are important considerations for overcoming the limitations of clinical trials to develop effective PAI-1-based therapeutics. Extensive further research will be needed for the successful development of PAI-1 therapeutics to treat severe human diseases.

Our recent studies reenforce the clinical significance of PAI-1 and its role in oncogenesis of hepatocytes. In particular, the inhibitory effect of PAI-1 in liver fibrosis/HCC appears to be evident. Given the high level of PAI-1 expression and its pathogenic role in fibrosis, the development of therapeutic agents to modulate PAI-1 signaling pathways and specific molecules targeting for regulating PAI-1 expression would provide promising anti-fibrotic strategies.

## Abbreviations

PAI-1: Plasminogen Activator Inhibitor-1
CSC: Cancer Stem-like Cells
HCV: Hepatitis C Virus
HSC: Hepatic Stellate Cells
EMT: Epithelial-mesenchymal Transition
ECM: Extracellular Matrix
DAA: Direct-acting Antiviral
